# Culturally inclusive education for international nursing students in Australia - barriers, educator preparedness and inclusive teaching strategies :An integrative review

**DOI:** 10.1016/j.ijnsa.2026.100697

**Published:** 2026-10-04

**Authors:** Daniel Johnson, Amanda Graf, Vasiliki Betihavas

**Affiliations:** a School of Nursing & Midwifery, University of Notre Dame Australia, Australia; b School of Nursing, Institute of Health and Nursing Australia (IHNA), Melbourne, Australia; c Campbelltown Hospital, (Campbelltown Emergency Department), NSW, Australia; d School of Nursing & Midwifery, Edith Cowan University, Perth, Australia

**Keywords:** Cultural inclusivity, Teaching practices, Australia, Nurse education, CALD, Curriculum, Andragogy

## Abstract

**Aim:**

This review explored and described the state of cultural inclusivity in teaching practices within Australian nursing programs, with particular attention to how those practices shaped the educational experiences of international and other culturally and linguistically diverse nursing students.

**Background:**

Nursing programs in Australia serve increasingly culturally diverse student cohorts. Cultural inclusion has been a long-standing concern in Australian nursing education, with growing numbers of international and other culturally and linguistically diverse students over the past two decades. Australian qualitative evidence indicates that teaching and learning environments can privilege Western educational and communication norms. Inclusivity is central to student engagement, readiness for practice, and patient safety.

**Design:**

An integrative review design was adopted to synthesise qualitative evidence related to cultural inclusivity in Australian nursing education.

**Methods:**

Eligibility permitted qualitative, quantitative and mixed methods empirical studies; all studies meeting the criteria were qualitative. No date limit was applied, which traced how cultural inclusion for international nursing students in Australia evolved over time. Four databases were searched. Eight qualitative studies were included, appraised using the Critical Appraisal Skills Programme Qualitative Studies Checklist, and analysed using Braun and Clarke’s reflexive thematic analysis.

**Results:**

Analysis of the eight qualitative studies produced five interconnected themes: language and communication barriers; social inclusion challenges and discrimination; educator unpreparedness; curriculum and support limitations; and inclusive teaching strategies. Two themes contained a distinct sub-theme, gendered cultural stigma within Theme 2 and pre-placement readiness within Theme 4. Across the included studies, attention to cultural inclusivity was uneven across academic and clinical learning contexts.

**Conclusions:**

Available qualitative evidence suggests that culturally inclusive nursing education requires coordinated educator development, explicit curriculum design, structured student support, and inclusive clinical-learning partnerships. The findings support a shift from reliance on individual educator initiative towards consistent institutional expectations, with local evaluation of any strategies adopted.

**What is already known**

• International nursing students in Australia can experience language and communication barriers, social exclusion and discrimination across academic and clinical learning settings.
• Cultural diversity is recognised within Australian nursing education standards, yet culturally inclusive teaching is not consistently embedded in curricula, assessment or placement support.

**What this paper adds**

• This integrative review synthesises eight Australian qualitative studies published between 2008 and 2025, showing that barriers to cultural inclusion have persisted over time.
• The review identifies five interconnected areas requiring attention: language and communication, social inclusion and discrimination, educator preparedness, curriculum and support systems, and inclusive teaching strategies.
• Findings indicate that culturally inclusive nursing education requires coordinated institutional action, including educator development, explicit curriculum integration, structured pre-placement and placement support, and local evaluation of strategies.

## 1 Introduction

Australia is widely recognised as a culturally diverse nation. The 2021 Census reveals that over one-quarter of the population was born overseas, and many speak a language other than English at home (Australian Bureau of Statistics, 2021). The nursing workforce reflects this pattern, with a significant proportion of newly registered nurses educated outside Australia (Nursing and Midwifery Board of Australia, 2023). This demographic context means that cultural diversity is central to nursing education, and national regulators expect programs to embed cultural safety, inclusion, and respectful practice within curricula (Australian Nursing and Midwifery Accreditation Council, 2025; Nursing and Midwifery Board of Australia, 2019).

Cultural inclusivity for international nursing students in Australia is not a new concern. International student enrolments in Australian nursing programs have grown steadily over the past two decades, and the challenges these students face have been discussed in the education literature for well over ten years. Parker and McMillan (2008) drew attention to the difficulties nursing academics encountered in teaching culturally diverse cohorts, and Jeong et al. (2011) documented the language and social barriers experienced by international nursing students. This early work described nursing education as carrying embedded Western-centred assumptions that disadvantaged culturally diverse learners. The focus has since sharpened, with more recent studies examining the experiences of international students in academic and clinical settings, yet the tensions identified in the earlier literature appear to remain unresolved.

Despite regulatory expectations, Australian qualitative evidence indicates that teaching in many nursing programs continues to reflect a Western-centred teaching philosophy (Graham et al., 2024; Jeong et al., 2011; Parker & McMillan, 2008). In this review, a Western-centred teaching philosophy refers to andragogy grounded in Anglo-European educational norms and communication styles, which positions these norms as neutral or default and treats other cultural ways of learning and interacting as departures from them. Cultural inclusivity requires teaching practices that recognise and integrate students’ cultural identities and learning needs, support participation and promote equitable engagement (Gay, 2018; Hockings, 2010). This aligns with the Australian Nursing and Midwifery Accreditation Council standard 3.7, which specifies that program content and subject learning outcomes must embed principles of diversity, culture, inclusion and cultural safety for all people (Australian Nursing and Midwifery Accreditation Council, 2025). Broader evidence indicates that implementation of such practices is inconsistent across levels of nursing education (Curtis et al., 2019). In some programs, culture is addressed only at a surface level or omitted, which can leave students feeling that their backgrounds and experiences are not recognised (Graham et al., 2024; Parker & McMillan, 2008).

Existing literature has identified shortcomings in cultural inclusivity within Australian nursing education. Parker and McMillan (2008) and Jeong et al. (2011) reported that educators often felt unprepared and that culturally and linguistically diverse students experienced language barriers, social isolation and limited support. More recent work by Graham et al. (2024) found that international students continued to encounter communication challenges, exclusion and racism in academic and clinical contexts.

Recent Australian studies indicate that the concern remains current. Lin et al. (2021) examined support for culturally and linguistically diverse undergraduate nursing students on clinical placement and found that clinical facilitators needed structured resources and strategies to teach these students effectively. Hari et al. (2021) reported that clinical supervisors adapted their teaching style, orientation and preceptor arrangements to support students from culturally and linguistically diverse backgrounds but did so without consistent institutional guidance. Gao et al. (2025) found that clinical educators of non-native English-speaking international nursing students identified persistent communication and assessment challenges and called for clearer teaching and support practices during first placements. Ghimire and Qiu (2025) reported that male South Asian international nursing students navigated cultural stigma around men in nursing and used linguistic strategies to manage family and community judgment. A further study, McCullough et al. (2024), described the creation of a dedicated coordinator role to support international nursing students; it was retained as contextual evidence rather than an included study because its cohort combined nursing and midwifery students. Taken together, this evidence indicates that educator preparation, curriculum design and institutional support continue to be identified as areas requiring attention.

These studies provide important insights, but no integrative review has specifically examined cultural inclusivity in teaching practices within Australian nursing programs. As a result, there is limited synthesised knowledge of how educators and institutions embed inclusive andragogy. This shortfall restricts the ability of programs to align teaching with national standards and to respond to the needs of diverse student cohorts.

This review addresses that shortfall by synthesising Australian qualitative evidence on cultural inclusivity in nursing education. Vygotsky’s sociocultural theory provides a lens for analysing how learning is shaped by interaction, language and cultural environment (Vygotsky, 1978). Using this lens, the review contributes to the development of more culturally inclusive approaches that promote equitable participation and culturally relevant learning.

For the purpose of this review, cultural inclusivity refers to teaching approaches that deliberately recognise, value and integrate the cultural identities and learning needs of all students so that each learner can participate equitably in academic, simulation and clinical environments. Cultural awareness involves recognising that cultural differences exist and acknowledging how these differences affect communication and interaction. Cultural inclusivity extends this awareness by actively reshaping teaching practices, learning environments and educator responses so that diverse students are not only acknowledged but meaningfully supported. In this way, cultural awareness becomes a precursor to inclusivity, because knowledge of culture is needed before educators can adjust andragogy and create learning spaces where all students feel respected and able to contribute.

## 2 Methods

### 2.1 Design

This review follows Whittemore and Knafl’s (2005) five-stage framework, which accommodates the inclusion of various research methodologies to provide a comprehensive exploration of a phenomenon. Eligibility criteria were intentionally broad with respect to study design. The evidence set that met the criteria was, however, exclusively qualitative, so the review is reported as an integrative review of qualitative evidence. The Whittemore and Knafl stages structured problem identification, literature search, data evaluation, data analysis and presentation, while analysis within stage four used Braun and Clarke’s six-phase reflexive thematic analysis (Braun & Clarke, 2006, 2019, 2021). This combination is appropriate because reflexive thematic analysis treats coding as an interpretative, author-led process rather than a reliability exercise, which suits the interpretive intent of the review question. Reporting follows the Preferred Reporting Items for Systematic Reviews and Meta-Analyses (PRISMA) 2020 statement where applicable to a review of qualitative evidence (Page et al., 2021).

### 2.2 Aim

This integrative review aimed to explore and describe the state of cultural inclusivity in teaching practices within Australian nursing programs, with particular attention to how these practices shape the educational experiences of international and other culturally and linguistically diverse nursing students.

### 2.3 Eligibility criteria

Inclusion and exclusion criteria were defined so that each criterion states a distinct condition and the criteria do not overlap. Studies were included if they met all of the following conditions:

• Setting: conducted in Australia, in a university or registered training organisation delivering nursing education.
• Population: international nursing students enrolled in a nursing program in Australia, and the nurse educators who teach and facilitate them. Studies of culturally and linguistically diverse nursing students were included where the cohort comprised, or substantially included, international students.
• Focus: teaching practices or strategies that promote cultural inclusivity within nursing education.
• Qualification level: diploma, bachelor or postgraduate nursing qualifications.
• Design: qualitative, quantitative or mixed methods studies, and theoretical or conceptual analyses consistent with integrative review methodology.

Studies were excluded if they met any of the following conditions:

• Primary focus on teaching students’ cultural competency to care for culturally diverse patients, rather than on inclusive teaching of culturally diverse students.
• Non-empirical opinion pieces or editorials with limited methodological rigour.
• Sources not available in English.

Inclusion and exclusion criteria ensured that this review maintained relevance, focus and methodological integrity.

No date limit was applied to the searches. This wide date range was a deliberate choice. The cultural inclusivity of international nursing students in Australia is a longstanding concern, and retaining older studies alongside recent ones allows the review to show how the problem has been discussed and addressed over more than a decade. The publication dates of the included studies are reported in Table 1, and the evolution of the problem across this range is examined in the analysis and discussion.

**Table 1:** Summary of included studies’ characteristics.

Study	Methodology	Participants	Aim	Key Results
Parker & McMillan (2008). Beyond Barriers: The Challenge of Cultural Diversity for Nurse Academics in the Australian Context	Critical ethnography with two phases (focus groups and interviews with academics, plus document review and class observation).	30 participants (27 nurse academics in five focus groups and 3 interviewed individually) from two schools of nursing in NSW, Australia.	To examine factors influencing the incorporation of cultural diversity in nursing curricula and how teaching and learning processes give voice to culturally different perspectives.	Teachers reported tensions from a lack of shared curricular philosophy, minimal attention to diversity, narrow views of community as a curriculum driver and reluctance to address difficult diversity issues in classrooms.
Jeong et al. (2011). Understanding and Enhancing the Learning Experiences of Culturally and Linguistically Diverse Nursing Students in an Australian Bachelor of Nursing Program	Qualitative exploratory pilot study using focus group interviews with thematic analysis.	18 participants (11 CALD students, 3 clinical facilitators and 4 academic staff) from a Bachelor of Nursing program.	To explore factors impeding or enhancing CALD students’ learning and staff teaching experiences and to identify support structures.	Four themes emerged: English language competence, feelings of isolation, limited learning opportunities and inadequate university support.
Graham et al. (2024). Experiences of International Nursing Students in a Regional University: A Clear Direction for Nursing Education	Qualitative design with six focus groups clustered by ethnicity (South Asian, Nepalese, Indian, multi-ethnicity). Constant comparative analysis to saturation.	17 international Bachelor of Nursing students from a regional Queensland university.	To explore cultural, societal and learning experiences of international nursing students and identify support needs in clinical placements.	Eight themes identified: preparedness to study, communication challenges, learning barriers, face-to-face learning contexts, bias and racism, university and community support, and personal strategies. Participants reported racism and communication difficulties in clinical settings and called for cultural safety training for educators and supervisors.
Abu-Arab & Parry (2015). Supervising Culturally and Linguistically Diverse (CALD) Nursing Students: A Challenge for Clinical Educators	Mixed questionnaire survey of two groups (educators and students) analysed using Yoder’s instructional responses framework.	Group 1: 8 female clinical educators (≥ 2 years experience). Group 2: 19 CALD nursing students (13 international, 6 local residents whose English is not their native language).	To identify difficulties encountered by clinical educators working with CALD students and suggest strategies to address them.	Educators struggled to respond to CALD students without being patronising or assimilationist and needed targeted pedagogical support. Students cited issues with fast speech and slang on wards and difficulty remembering drug names. Cross-cultural training and peer buddy systems were recommended.
Lin, Del Fabbro, Needham, Sidwell & Shaw (2021). Supporting culturally and linguistically diverse (CALD) undergraduate nursing students undertaking clinical placements in Australia: An exploratory qualitative study of clinical facilitator and CALD student perceptions	Qualitative study informed by cultural-historical activity theory; focus groups and individual interviews with constant comparative analysis; NVivo.	21 clinical facilitators (three focus groups and five individual interviews) and 13 CALD Bachelor of Nursing students (two focus groups; 85% international; India, Hong Kong, China, Korea, Japan, Nepal, New Zealand) at a university in southeast Queensland, Australia.	To explore clinical facilitators’ perceptions of supervising CALD nursing students and the factors shaping the clinical learning environment.	Facilitators described language and communication barriers, differing cultural norms around questioning and hierarchy, and the additional time required to support CALD students. Preparation and resources for facilitators were limited, and facilitators wanted structured guidance to supervise CALD students effectively.
Hari, Geraghty & Kumar (2021). Clinical supervisors' perspectives of factors influencing clinical learning experience of nursing students from culturally and linguistically diverse backgrounds during placement: a qualitative study	Descriptive qualitative study; semi-structured interviews; thematic analysis; data collected 2017.	9 clinical supervisors (6 clinical educators and 3 preceptors; 5 overseas-born) at one large public hospital in the Northern Territory, Australia.	To explore clinical supervisors’ experiences of supervising CALD nursing students during clinical placement.	Three themes: Communication, Culture and Contextual factors. Supervisors identified English proficiency and accent as barriers, cultural norms around hierarchy that discouraged students from speaking up, and the influence of gender and family expectations. Time and workload limited the support supervisors could provide.
Gao, Madhuvu & Shimoinaba (2025). Clinical educators and non-native English-speaking nursing students in first clinical placements: A qualitative exploration	Qualitative descriptive design; semi-structured interviews; reflexive thematic analysis (Braun & Clarke); NVivo v20; interviews September 2022 to January 2023.	10 non-native English-speaking first-year international Bachelor of Nursing students (4 female, 6 male; China 6, India 1, Malaysia 1, Singapore 1, Vietnam 1) and 11 clinical educators at a university in Victoria, Australia.	To explore the experiences of non-native English-speaking international nursing students and their clinical educators during first clinical placements.	Students reported uncertainty, language and cultural challenges and assessment concerns during first placement. Educators recognised these challenges and suggested clearer communication, cultural awareness and structured support. Both groups highlighted the need for preparation before placement.
Ghimire & Qiu (2025). “I call it health science, not nursing”: Male international students balancing nursing career aspirations with cultural stigma	Qualitative descriptive design; semi-structured interviews; thematic analysis (Braun & Clarke) informed by intersectionality, identity work and transnationalism; COREQ; member-checking.	11 male South Asian international nursing students in the final semester of a three-year Bachelor of Nursing, aged 23–27, at a university in Victoria, Australia.	To explore how male South Asian international nursing students navigate cultural stigma and identity while pursuing nursing careers in Australia.	Three themes: cultural stigma around men in nursing that led students to describe their course as “health science”; migration, masculinity and family expectations shaping career choices; and linguistic strategies to manage stigma. Students concealed the “nurse” label to manage family and community judgement.

### 2.4 Search strategies

The search strategy was developed in consultation with a research librarian to ensure methodological rigour. Four electronic databases were searched: Cumulative Index to Nursing and Allied Health Literature (CINAHL), MEDLINE, Scopus and ProQuest Central. These databases were selected for their coverage of nursing, health and education literature and together, span the biomedical, allied health and social science indexing relevant to the review question.

Search concepts were combined across three domains: cultural inclusivity, nursing education and the Australian setting. Within each domain, controlled vocabulary and free-text terms were combined with the Boolean operator OR, and the three domains were then combined with AND. An indicative CINAHL string was: (“cultural inclusiv*” OR “cultural safety” OR “culturally responsive” OR “culturally and linguistically diverse” OR CALD OR multicultural) AND (nurs* AND (educat* OR curriculum OR teaching OR student* OR "clinical placement")) AND (Australia*). Truncation and database-specific subject headings were adapted for each database. Searches were restricted to peer-reviewed articles published in English. Reference lists of included studies were hand-searched to identify further sources.

### 2.5 Screening and selection

Title and abstract screening were conducted independently by two authors against the eligibility criteria. Full-text articles were then retrieved and assessed against an eligibility matrix. Disagreements about inclusion were resolved by discussion, with a third author consulted where consensus could not be reached. Study selection was therefore a two-reviewer process with a third reviewer resolving disagreements. This is distinct from the interpretative coding reported under Data synthesis, which was a single-analyst reflexive process. The selection process is summarised in the PRISMA flow diagram (see Fig. 1).

**Fig. 1:**
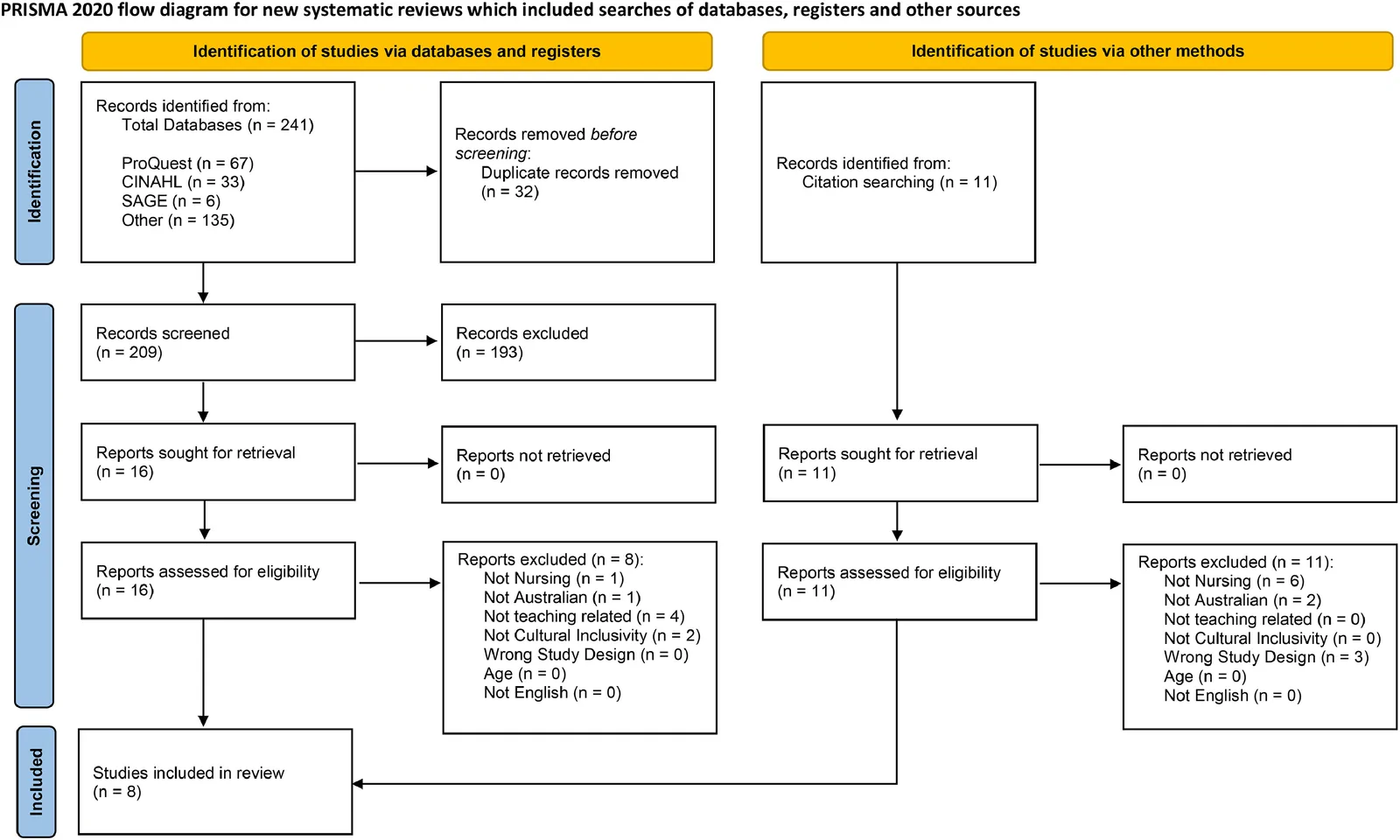
PRISMA flow diagram (Page et al., 2021).

### 2.6 Critical appraisal

Included studies were critically appraised using the Critical Appraisal Skills Programme (CASP) Qualitative Studies Checklist (Critical Appraisal Skills Programme, 2018). The checklist was selected because all eight included studies used qualitative designs, and the tool appraises the aim, methodology, research design, recruitment strategy, data collection, the relationship between researcher and participants, ethical considerations, analytical rigour, clarity of findings and value of the research. Consistent with an interpretive synthesis, appraisal was used to inform the weight given to each study rather than to exclude studies, and no overall numerical quality score was calculated. Responses to each item were recorded as yes, partial or can't tell. Across the eight included studies, most criteria were met. The criterion least consistently met was consideration of the relationship between researcher and participants, with several studies providing a limited reflexive account of researcher influence on the findings.

A qualitative-specific checklist was appropriate because the eligibility criteria permitted qualitative, quantitative and mixed methods designs, and all eight studies that met the criteria were qualitative. The CASP checklist provided consistent, criterion-referenced appraisal that allowed each study to be assessed against recognised standards for qualitative research. Studies that relied on a single analyst, or that reported limited detail of their coding process, were given proportionately less interpretive weight where they were the sole source for a theme.

### 2.7 Data extraction

A table (Table 1) was created summarising each study, including author, year, setting, participants, study design and main findings relevant to cultural inclusivity in teaching practices in Australian nursing programs. The eligibility criteria permitted qualitative, quantitative and mixed methods studies; all eight studies that met the criteria were qualitative. The major themes and quotations from students and academics were extracted. Data extraction was conducted by the primary author.

### 2.8 Data synthesis

Findings were synthesised using Braun and Clarke’s six-phase reflexive thematic analysis (Braun & Clarke, 2006, 2019, 2021), applied inductively across the full texts of the included studies. While study selection involved two reviewers, interpretive coding was undertaken by the primary author as a single-analyst reflexive process; the six phases comprised familiarisation, generating initial codes, constructing candidate themes, reviewing themes against the coded data and the full dataset, defining and naming themes, and producing the synthesis. Rather than seeking inter-coder agreement, which is not consistent with a reflexive approach, analytical decisions were subjected to ongoing reflexive questioning by the primary author, and each stage was documented in an audit trail to support transparency. The primary author’s positionality, described below, was treated as an analytical resource within this process.

### 2.9 Positionality of the primary author

The primary author is a nurse educator who teaches culturally diverse cohorts in Australian nursing programs. This experience supports the interpretation of the review’s findings by grounding it in practical knowledge of the day-to-day realities involved in supporting international nursing students across classroom and simulation environments. The author recognises that their perspectives are shaped by their professional background within the Australian vocational and higher education sectors and acknowledges the potential for these experiences to influence the analysis. To maintain reflexivity, the primary author engaged in ongoing critical reflection and documented the audit trail to make interpretive decisions transparent and open to scrutiny. This positionality is not presented as a limitation, but as a contextual factor that strengthens the authenticity and relevance of insights into culturally inclusive teaching practices (Brookfield, 2017).

## 3 Results

### 3.1 Study characteristics

Eight qualitative studies published between 2008 and 2025 met the eligibility criteria. All were conducted in Australian university nursing programs. Participants included nursing students, nursing academics, clinical educators, clinical facilitators and clinical supervisors. Study characteristics are summarised in Table 1, and the basis on which each study addressed the review question, is set out in Table 2.

**Table 2:** How the included studies aligned with the review aim.

Author	Alignment
Parker & McMillan (2008)	Nurse academics reported insufficient curricular commitment to cultural diversity and limited andragogical adaptation to multicultural classrooms. There was a lack of shared philosophy about inclusive curricula, minimal exploration of racism or discrimination in learning environments, and avoidance of conflictual diversity issues. The results demonstrated that Australian nursing programs often treat culture as peripheral rather than integral to teaching design.
Jeong et al. (2011)	CALD students experienced language barriers, isolation and restricted learning opportunities, while university support and staff cultural awareness were inadequate. The results exposed an absence of systematic approaches to culturally inclusive teaching and the need for staff capability-building to address intercultural learning barriers and ensure equitable engagement in clinical and classroom settings.
Graham et al. (2024)	International nursing students described communication challenges, bias and racism within both theoretical and clinical learning spaces. Educators and supervisors lacked cultural safety competence, limiting inclusive practice. Students sought greater preparation and culturally responsive teaching to adapt to Australian critical-thinking and participation expectations. The findings highlight persistent shortcomings in inclusive andragogy and call for structured cultural safety professional development across curricula.
Abu-Arab & Parry (2015)	Clinical educators supervising CALD students reported difficulty applying equitable teaching responses without appearing assimilationist or patronising. Many attributed problems to students’ English proficiency rather than systemic teaching limitations. The results showed educators’ limited intercultural competence and institutional support, signalling the need for explicit inclusive supervision models, cross-cultural communication training and culturally informed feedback mechanisms in nursing education.
Lin et al. (2021)	Clinical facilitators supervising CALD students described language and communication barriers, cultural norms around questioning and hierarchy, and the extra time required to support these students. Facilitators reported limited preparation and resources, reinforcing the review’s findings on educator unpreparedness and the need for structured supervision guidance in the clinical learning environment.
Hari et al. (2021)	Clinical supervisors identified English proficiency and accent as communication barriers and described cultural norms around hierarchy that discouraged students from speaking up. Gender and family expectations, together with time and workload pressures, shaped the support available. The findings align with the review’s themes on language barriers, social inclusion and contextual constraints on inclusive supervision.
Gao et al. (2025)	Non-native English-speaking first-year international students and their clinical educators both reported language and cultural challenges, assessment concerns and uncertainty during first placement. Educators acknowledged limited preparation and recommended pre-placement support. The study aligns with the review’s themes on communication barriers, curriculum and support limitations, and inclusive teaching strategies.
Ghimire & Qiu (2025)	Male South Asian international students navigated cultural stigma around men in nursing, concealing the “nurse” label to manage family and community judgment. Migration, masculinity and family expectations shaped their choices. The study extends the review’s theme on social inclusion and discrimination by showing how gendered cultural stigma intersects with the international student experience.

### 3.2 Key themes

Initial codes were generated inductively from the full texts of the eight included studies. Open coding produced 111 initial codes, which were reviewed and consolidated into a smaller set of grouped codes and organised into a final framework of five themes. Two themes each contain a distinct sub-theme: a gendered cultural stigma sub-theme within Theme 2 and a pre-placement readiness sub-theme within Theme 4. Theme labels were refined during the defining and naming phase to reflect their scope and meaning. The contributing studies for each theme and sub-theme are recorded in Table 3 as illustrative codes, and the themes are read across the publication dates of the included studies so that the persistence of these concerns is visible within each theme.

**Table 3:** Illustrative Coding framework.

Initial Codes / Data Fragments	Domain	Sub-theme	Theme	Source(s)
“It is often difficult to know what the [CALD] student’s comprehension and/or understanding related to patient care is in the clinical setting.”	Teaching & Communication	Limited English proficiency and comprehension difficulties	Language & Communication Barriers	Abu-Arab & Parry (2015).
“NESB [non-English speaking background] students tend to just nod in agreement if you are trying to explain something.”	Teaching & Communication	Passive communication behaviours	Language & Communication Barriers	Abu-Arab & Parry (2015).
“Somebody will say you are not communicating well with your English… I can’t communicate… she doesn’t want to listen.”	Student–Educator Interaction	Miscommunication and accent difficulties	Language & Communication Barriers	Jeong et al. (2011).
“The medium of instruction is English but still the problem is we don’t communicate much in English. We use our [own] languages.”	Classroom Environment	Intragroup language use reducing integration	Language & Communication Barriers	Graham et al. (2024).
“It is so time consuming. Some of the students…have trouble with [language].”	Teaching & Communication	Time cost of language support	Language & Communication Barriers	Lin et al. (2021).
“I can’t teach English in 4 weeks or professional English in 4 weeks… then I think we are already on the backfoot.”	Clinical Placement	English proficiency limits within placement time	Language & Communication Barriers	Hari et al. (2021).
Students felt “the English spoken by patients did not align with the ‘Standard English’ they had learned”.	Clinical Placement	Real-world versus textbook English	Language & Communication Barriers	Gao et al. (2025).
“Social isolation of CALD students was exacerbated by the rejection and discrimination they experienced from domestic students.”	Learning Environment	Peer exclusion and lack of belonging	Social Inclusion Challenges & Discrimination	Jeong et al. (2011).
“Basically bullying… in debriefing sessions… other students said ‘spit it out’.”	Learning Environment	Bullying and microaggressions	Social Inclusion Challenges & Discrimination	Jeong et al. (2011).
“A colleague told me, I don’t like Indians… she was laughing at my accent.”	Clinical Placement	Racial bias and humiliation	Social Inclusion Challenges & Discrimination	Graham et al. (2024).
“Perceived as being at the bottom of the pecking order, and so it’s not their right to speak out.”	Cultural Norms	Hierarchy and reluctance to speak up	Social Inclusion Challenges & Discrimination	Hari et al. (2021).
“In my country, nursing is seen strictly as a woman’s job.” “By calling it ‘health science,’ I preserve their respect and can pursue my ambitions.”	Social / Cultural Identity	Sub-theme: Gendered cultural stigma and identity concealment	Social Inclusion Challenges & Discrimination	Ghimire & Qiu (2025).
“I had an experience with a male [CALD] student, who found it very difficult taking instruction from a female.”	Cultural Norms	Sub-theme: Gendered cultural stigma and identity concealment	Social Inclusion Challenges & Discrimination	Hari et al. (2021).
“Seven out of the eight clinical educators expressed difficulty working with CALD students and none of them considered they were very well prepared.”	Educator Competence	Low self-rated readiness for diversity	Educator Unpreparedness	Abu-Arab & Parry (2015).
“Some Australian nurse teachers are not well prepared for the challenges they face, nor have nursing curricula been re-conceptualised.”	Academic Staff Development	Lack of professional development and cultural competence training	Educator Unpreparedness	Parker & McMillan (2008).
“CALD students acknowledged the challenges … could have been lessened by a more structured and supportive approach.”	Institutional Support	Lack of structured orientation and guidance	Curriculum & Support Limitations	Jeong et al. (2011).
Cultural content in nursing was “often overlooked, minimalistic, tokenistic”.	Curriculum Design	Superficial cultural content	Curriculum & Support Limitations	Graham et al. (2024).
“Teachers reported tensions arising from lack of a shared philosophical view about curricula … and a reluctance to address diversity issues.”	Institutional Practice	Weak institutional framework for diversity	Curriculum & Support Limitations	Parker & McMillan (2008).
“I’d expect them to be prepared prior to placement, making sure the pre-placement documents are done and completed, and submitted.”	Institutional Support	Sub-theme: Pre-placement readiness and preparation	Curriculum & Support Limitations	Gao et al. (2025).
“I would like to meet the educator herself, or maybe meet her on a Zoom meeting … get myself familiar with this person who I’ll be seeing for the two weeks.”	Institutional Support	Sub-theme: Pre-placement readiness and preparation	Curriculum & Support Limitations	Gao et al. (2025).
“Structured role plays to practise nursing terminology and improve communication.”	Teaching Innovation	Applied language practice and simulation	Inclusive Teaching Strategies	Abu-Arab & Parry (2015).
“Pairing CALD students with Australian-born peers for conversation practice.”	Peer Learning	Buddy and mentoring programs	Inclusive Teaching Strategies	Abu-Arab & Parry (2015).
“Cross-cultural communication workshops for educators and students.”	Professional Development	Cultural safety and awareness training	Inclusive Teaching Strategies	Abu-Arab & Parry (2015).

Theme 1 **Language and Communication Barriers**

This theme addresses difficulties in English language proficiency and communication that impeded learning and interaction. It was supported by six of the eight included studies and spans the full publication range.

Clinical instructors in Abu-Arab and Parry’s (2015) study reported, “It is often difficult to know what the student’s comprehension and/or understanding related to patient care is in the clinical setting … It is very difficult as an educator to gauge what the students’ [expectations] are” (p. e4). Students with limited English would sometimes “just nod in agreement” (Abu-Arab & Parry, 2015, p. e5) even when they had not grasped the topic taught, which masked comprehension problems. Jeong et al. (2011) similarly found that language difficulties reduced classroom engagement, and reported that domestic peers in group projects voiced frustration, with one student saying, “I don’t want to work with an international student because I can’t talk to them and they can’t talk to me” (p. 241). Those peers also raised concerns about receiving a lower grade when paired with an international student.

International students also described their own language use. Graham et al. (2024) reported a student who noted that while “[the] medium of instruction is English” (p. 213), in practice “we don’t communicate much in English. We use our [own] languages” (p. 213). In nursing, where English proficiency is directly connected to safe practice, participants and authors interpreted this pattern as reducing opportunities to develop English skills.

Across these studies, communication difficulties co-occurred with reduced confidence and participation (Graham et al., 2024; Jeong et al., 2011).

More recent studies reinforced this theme. A clinical facilitator in Lin et al. (2021) described the time cost of language support, noting that “it is so time-consuming. Some of the students…have trouble with comprehension” (p. 4). In Hari et al. (2021), a supervisor observed the limits of correcting proficiency within a placement, stating, “I can’t teach English in 4 weeks or professional English in 4 weeks… then I think we are already on the backfoot” (CS1, p. 3). Gao et al. (2025) reported that non-native English-speaking students found that the English spoken by patients did not align with the “Standard English” they had learned (p. 144), which slowed communication during their first placement.

Theme 2 **Social Inclusion Challenges and Discrimination**

This theme addresses student experiences of social isolation, exclusion and discrimination that undermined a sense of belonging. It draws principally on Jeong et al. (2011), Graham et al. (2024) and Parker and McMillan (2008), with supporting material from Hari et al. (2021) and Ghimire and Qiu (2025).

Participants in the included studies described feeling marginalised within Australian education settings. Jeong et al. (2011) reported that international nursing students frequently experienced exclusion by local classmates, noting that “social isolation of Culturally and Linguistically Diverse (CALD) students was exacerbated by the rejection and discrimination they experienced from domestic students” (p. 241). Jeong et al. (2011) also described a clinical facilitator’s account of overt bullying during placement debriefings; when a diverse student struggled to express themselves, other students would taunt them to “spit it out, spit it out” (p. 241), behaviour the facilitator deemed “quite rude and inappropriate” (p. 241).

Graham et al. (2024) also reported bias and racism in both academic and clinical environments. Participants recounted being treated differently by supervisors and hearing openly racist remarks. One student said, “A colleague told me, ‘I don’t like Indians’” (p. 214) and recalled that “she was laughing at my accent” (p. 214).

From the educator perspective, participants described such behaviour as not consistently addressed. Parker and McMillan (2008) reported that many nursing academics felt ill-equipped or hesitant to confront the “difficult and contentious issues arising from diversity in the classroom” (p. 145), and that educators would often avoid frank discussions of discrimination.

A distinct sub-theme, gendered cultural stigma and identity concealment, extends this theme. Ghimire and Qiu (2025) reported that male South Asian students managed cultural stigma around men in nursing, with one participant explaining, “In my country, nursing is seen strictly as a woman’s job” (p. 585), and another describing linguistic camouflage: “By calling it ‘health science’, I preserve their respect and can pursue my ambitions” (p. 585). This stigma originates in the student’s own cultural and family context rather than in Australian classroom or clinical relationships, which distinguishes it from the discrimination described above. It is retained within this theme because participants described it as shaping their willingness to identify openly as nursing students and to engage with peers and educators, which is directly relevant to belonging within Australian nursing programs. Because this sub-theme rests on two studies, it is reported as an indicative finding that warrants further investigation rather than an established feature of Australian nursing education.

Theme 3 **Educator Unpreparedness**

This theme addresses limitations in educators’ training, confidence and readiness to teach and support a culturally diverse student cohort. It was supported by Parker and McMillan (2008), Abu-Arab and Parry (2015) and Lin et al. (2021).

Across the included studies, educator participants described limited preparation for teaching culturally diverse cohorts. In relation to supervising CALD students, none of the clinical educators surveyed reported feeling “very well prepared” to supervise these students, and “the great majority (seven out of eight) chose scores between 1 (not prepared) and 3 (moderately prepared)” on the preparedness scale (Abu-Arab & Parry, 2015, p. e6). Focus group participants described cultural diversity teaching as “subtle and needing to be seasoned, and yet we are expected to just do it” (Parker & McMillan, 2008, p. 147).

A more recent study reported the same pattern in clinical settings. Lin et al. (2021) found that clinical facilitators felt under-resourced to supervise culturally and linguistically diverse students and wanted structured guidance.

Theme 4 **Curriculum and Support Limitations**

This theme addresses shortcomings in nursing curricula and institutional support systems that limited the integration of cultural inclusivity into teaching practice. It draws principally on Parker and McMillan (2008), Jeong et al. (2011), Abu-Arab and Parry (2015) and Graham et al. (2024), with the sub-theme drawing on Gao et al. (2025) and Hari et al. (2021).

Participants in several studies described cultural inclusivity as peripheral rather than core to the curriculum. Parker and McMillan (2008) described an absence of a unifying commitment to diversity in curricular design. One focus group participant acknowledged that “we do avoid things that are in any way controversial, and we certainly do not address different cultures or different religious views in any depth at all” (Parker & McMillan, 2008, p. 146). Academics commented that imperatives to include cultural diversity “in the absence of shared understandings, clear direction and agreed-upon methods often lead to tokenistic inclusion of content”, with references to cultural diversity “reflected in broad statements of purpose” but with “often no translation into learning processes or assessment activities” (Parker & McMillan, 2008, p. 148). Students described limited culturally responsive support, reporting that “I just need more support from the teachers' ideas” when delivering learning activities (Graham et al., 2024, p. 214).

Participants also described limited institutional support. Jeong et al. (2011) noted that their participants faced initial obstacles in enrolment and learning that “could have been lessened by a more structured and supportive approach” (p. 241). In Abu-Arab and Parry’s (2015) study, educators highlighted limited support from both the university and clinical facilities in how to supervise diverse students.

A distinct sub-theme, pre-placement readiness and preparation, addresses needs and gaps specific to the period before placement begins, which is analytically separate from the in-placement resourcing described above. A clinical educator in Gao et al. (2025) expected students to be “prepared prior to placement, making sure the pre-placement documents are done and completed and submitted” (p. 143), while students in the same study asked to “meet the educator herself, or maybe meet her on a Zoom meeting” before placement (p. 142), and supervisors in Hari et al. (2021) noted that language readiness could not be addressed within a short placement (p. 3). Participants in these two studies described structured pre-placement support as assumed rather than provided. As with the sub-theme in Theme 2, this finding rests on a small number of studies and is reported as indicative.

Theme 5 **Inclusive Teaching Strategies**

The included studies also reported strategies proposed or trialled to support a more inclusive learning environment. None of these strategies was formally evaluated for effectiveness within the included studies, so they are reported as participant-identified or author-proposed approaches rather than as demonstrated interventions.

One approach was to create structured opportunities for skill-building in communication and interaction. Abu-Arab and Parry (2015) reported that educators suggested “structured role plays to practise nursing terminology and improve communication” (p. e7). Participants described such simulated scenarios as a way for students to gain confidence with health terminology in a lower-risk setting.

A second approach was peer pairing or peer mentoring. Abu-Arab and Parry (2015) found value in “pairing CALD students with Australian-born peers for conversation practice” (p. e7). A comparable arrangement was reported in Jeong et al. (2011), where the authors implemented a student mentor program in which senior students supported new culturally diverse students. Outcomes of that program were not reported in a form that allows conclusions about effectiveness.

A third approach was cross-cultural training and professional development. Abu-Arab and Parry (2015) called for cross-cultural communication workshops for educators and students, held outside the pressured clinical environment, with the aim of building mutual understanding and increasing educators’ cultural awareness. Lin et al. (2021) and Hari et al. (2021) similarly reported requests for structured resources, orientation and supervisor guidance. Graham et al. (2024) recommended cultural safety professional development for academics and placement supervisors.

### 3.3 Synthesis across themes

Read together, the five themes describe a learning environment in which language, belonging, educator readiness and curriculum systems interact. Across the included studies, language and communication difficulties co-occurred with social isolation and, in some accounts, with discriminatory behaviour by peers or staff. The evidence does not establish that language difficulty caused discriminatory behaviour. Educator participants described limited preparation for responding to these situations, and participants in several studies described curriculum and institutional arrangements that provided few consistent mechanisms for prevention or response.

Interpreted through Vygotsky’s sociocultural theory, these accounts indicate that culturally inclusive andragogy in the settings studied depends on multiple interacting conditions rather than on any single practice (Vygotsky, 1978). Where one condition is weak, participants described consequences across the others. Because the evidence base comprises eight qualitative studies from university settings, these patterns describe the accounts of the participants in those studies and should not be read as an estimate of practice across the Australian nursing education sector.

## 4 Discussion

The synthesis showed that cultural inclusivity in Australian nursing programs is inconsistently embedded across theoretical and clinical learning environments. Although diversity is acknowledged, many teaching practices continue to reflect Western-centred norms (Parker & McMillan, 2008). Across the included studies, students described language barriers, social isolation, bias and limited learning opportunities, while educators reported discomfort, uncertainty and limited preparation for teaching culturally diverse cohorts.

Read across their publication dates, the included studies indicate a persistent rather than a historical problem. Earlier work by Parker and McMillan (2008) and Jeong et al. (2011) identified unprepared educators, exclusion and a curriculum that treated culture as peripheral, and more recent studies describe the same underlying tensions in the experiences of international students (Gao et al., 2025; Ghimire & Qiu, 2025; Hari et al., 2021; Lin et al., 2021). This continuity across seventeen years of Australian qualitative research is the review’s principal contribution, and it supports treating cultural inclusivity as a current research priority rather than a settled question. It also indicates that the evidence base remains small relative to the size of the international student cohort, which is itself a finding worth reporting.

From the educator’s perspective, discomfort and uncertainty shaped responses to culturally and ethically sensitive situations. Parker and McMillan (2008) observed that cultural inclusivity was peripheral in curriculum design and that many educators hesitated to address diversity-related issues. Later studies of clinical supervision reported the same hesitancy alongside requests for structured guidance (Hari et al., 2021; Lin et al., 2021). Taken together, these accounts suggest that a shared approach to culturally inclusive teaching had not been established in the settings studied.

From the student perspective, findings from both Jeong et al. (2011) and Graham et al. (2024) pointed to challenges with communication, participation and institutional support. Students from diverse backgrounds described feeling isolated, having limited opportunities to contribute and experiencing difficulty communicating with peers and supervisors. Together, these accounts suggest that culturally inclusive teaching is not consistently practised and that students may not always receive equitable or sufficient support in academic or clinical environments.

Clinical learning environments added another layer of complexity. Abu-Arab and Parry (2015) reported that clinical educators were often uncertain about how to support diverse students without relying on deficit-based or assimilation-focused assumptions. Students described difficulty keeping pace with rapid speech, slang and clinical terminology, while educators acknowledged that they did not always feel adequately prepared to address these challenges. These findings suggest that cultural inclusivity concerns extend beyond the classroom and are shaped by the broader expectations and cultures present in both educational and workplace settings.

Interpreting these findings through Vygotsky’s sociocultural theory reinforces that learning is shaped by socially mediated interaction. Barriers related to language, belonging and unaddressed bias restrict participation within the zone of proximal development, which limits students’ development of clinical reasoning, communication and confidence. When communication breakdowns or cultural misunderstandings disrupt interaction, students cannot fully engage with learning tasks or internalise new knowledge. Creating culturally inclusive learning environments is therefore central to supporting the social and relational foundations of learning.

The findings also reflect broader discussions on the hidden curriculum, which considers how unspoken norms, power relations and expectations shape the learning experience (Noonan et al., 2024; Pittman & Vue, 2025; Raso et al., 2019). Quieter ways of communicating or signs of hesitation are sometimes mistaken for a lack of interest, which can strengthen deficit views of students from diverse backgrounds. The included studies indicate that these hidden influences work alongside formal teaching practices, affecting how students are perceived and how educators interpret and respond to cultural differences.

Across the included studies, culturally inclusive andragogy appeared to rely largely on the commitment and confidence of individual educators rather than on consistent institutional systems. Limitations in educator readiness, uneven attention to culture within the curriculum and inconsistent responses to discrimination were each described by participants. On this evidence, improvement is likely to depend on sustained educator development, clear institutional expectations and curriculum structures that treat cultural inclusivity as a core educational principle rather than an occasional addition.

The strategies identified across the included studies provide a starting point rather than a proven program of change. Structured communication practice, peer support models and cross-cultural learning activities were valued by participants, but none was evaluated for effectiveness in the included studies. Their adoption should therefore be accompanied by local evaluation of participation, acceptability, equity and any unintended effects.

## 5 Implications for practice

### 5.1 Educator development

Professional development for educators should move beyond basic compliance and instead involve continual guidance in intercultural communication, cultural norms and reflective teaching. Educators need the capability to manage culturally sensitive discussions, recognise implicit bias (Gutiérrez & Rogoff, 2003; Sue, 2010) and adjust their teaching to suit a range of learning styles (Gay, 2018).

### 5.2 Curriculum design

Cultural inclusivity could be integrated through learning outcomes, teaching materials and assessment practices rather than treated as an adjunct topic, with explicit consideration of inclusive practice in program evaluation.

Universities could embed cultural inclusion indicators within learning outcome reviews and academic quality assurance processes so that inclusive teaching practices are applied consistently across units.

Registered training organisations could align culturally inclusive teaching practices with training package requirements and structured assessor moderation to support consistency across theory, simulation and workplace assessment in diploma of nursing delivery. This suggestion extends beyond the included evidence, which was drawn from university settings, and should be treated as a proposition for testing.

### 5.3 Institutional support

Structured student support, orientation tailored to diverse learners, peer mentoring programs, accessible academic skills assistance, and clear anti-discrimination policies should accompany curriculum changes.

Institutions would benefit from implementing routine monitoring of cultural safety outcomes by using student feedback, incident reporting trends and periodic review of teaching and placement experiences to evaluate whether culturally inclusive practices are being enacted consistently.

### 5.4 Clinical education partnerships

Clinical supervisors would benefit from receiving preparation in cultural safety and inclusive supervision. Structured communication scaffolds and cross-cultural workshops may enhance placement experiences and reduce discriminatory interactions.

### 5.5 Student engagement

Formal peer mentoring programs pairing experienced and new diverse students, and cross-cultural communication workshops bringing students and educators together, should be considered as standard program components.

Together, these implications outline a path towards strengthening inclusive teaching as a shared responsibility rather than an individual choice.

## 6 Limitations

The inclusion and exclusion criteria were applied during the title, abstract and full-text screening stages. Eight studies met all criteria. This modest number reflects the limited volume of Australian research that directly examines cultural inclusivity in nursing teaching, which affects the breadth of evidence that can be synthesised. The evidence base is also concentrated in a small number of studies, and some analytical elements, including the two sub-themes, draw substantially on individual studies; the findings should be read with this concentration in mind.

A further limitation relates to differences between the vocational and higher education settings. The included studies were based in university programs, which may not reflect the teaching practices, regulatory requirements or resource limitations common in the Australian vocational sector. Additionally, the included studies examined undergraduate programs and not postgraduate programs. This means the findings may not fully represent the broader landscape of culturally inclusive teaching across all nursing education pathways.

The review did not apply an intersectional lens. Current evidence shows that student experiences are influenced not only by culture and language, but also by the combined effects of migration status, socioeconomic position, gender and racialisation. Including an intersectional approach in future research would provide a more detailed understanding of how structural inequities shape learning for different groups of students.

## 7 Conclusion

This integrative review explored the current state of cultural inclusivity in teaching practices within Australian nursing programs and found that cultural inclusivity is not yet consistently embedded across classroom and clinical learning settings. While diversity is recognised at a conceptual level, many teaching approaches still draw heavily on Western educational norms, which can lead to uneven and sometimes inequitable learning experiences for students from culturally diverse backgrounds (Gay, 2018; Noonan et al., 2024; Pittman & Vue, 2025).

The review found continuing limitations in educator readiness, limited integration of culture within the curriculum and uneven responses to discrimination. These issues show that culturally inclusive andragogy often relies more on the initiative of individual educators than on coordinated institutional systems. Sustained implementation of culturally inclusive teaching practices will require continued development of educator capability, clearer institutional expectations and active support for culturally safe practice. It will also depend on curriculum structures that weave inclusivity through teaching materials, teaching approaches and assessment, ensuring it is part of everyday practice rather than an occasional focus.

Future research should examine how culturally inclusive teaching approaches are implemented and experienced across both vocational and higher education nursing settings. Studies should also investigate how institutional structures, clinical-learning partnerships and intersectional factors, including migration status, socioeconomic position, gender and racialisation, shape cultural safety in classroom and clinical environments. Further inquiry into these issues is warranted. Meaningful progress will require collaboration among educators, institutional leaders, students and clinical partners to ensure that teaching practices align with regulatory expectations and support fair, respectful and equitable participation for all learners.

## CRediT authorship contribution statement

**Daniel Johnson:** Writing – review & editing, Writing – original draft, Visualization, Validation, Software, Resources, Project administration, Methodology, Investigation, Formal analysis, Data curation, Conceptualization. **Amanda Graf:** Writing – review & editing, Visualization, Validation, Supervision, Resources, Project administration, Methodology, Investigation, Conceptualization. **Vasiliki Betihavas:** Writing – review & editing, Visualization, Validation, Supervision, Resources, Project administration, Methodology, Investigation, Conceptualization.

## Declaration of competing interest

The authors declare that they have no known competing financial interests or personal relationships that could have appeared to influence the work reported in this paper.
